# Novel Molecular Subtypes Associated With 5mC Methylation and Their Role in Hepatocellular Carcinoma Immunotherapy

**DOI:** 10.3389/fmolb.2020.562441

**Published:** 2020-10-23

**Authors:** Zhuomao Mo, Zhirui Cao, Shaoju Luo, Yan Chen, Shijun Zhang

**Affiliations:** ^1^Department of Traditional Chinese Medicine, The First Affiliated Hospital, Sun Yat-sen University, Guangzhou, China; ^2^Department of Traditional Chinese Medicine, The Third Affiliated Hospital, Sun Yat-sen University, Guangzhou, China

**Keywords:** hepatocellular carcinoma, molecular subtype, score, immunotherapy, 5mC methylation

## Abstract

**Background:**

5-methylcytosine (5mC) has been reported in the prognosis of a variety of cancers, however, its role in hepatocellular carcinoma (HCC) has not been investigated yet. This study aimed at identifying the molecular subtypes associated with 5mC and establishing a relevant score to predict its prognosis in HCC.

**Methods:**

Somatic gene mutation data and gene expression data were retrieved from The Cancer Genome Atlas database. Molecular subtypes were identified by unsupervised clustering based on the expression of 5mC regulators, and the molecular features of each subtype were investigated by survival, mutation, gene set variation, and immune cell infiltration analyses. Next, we performed a differentially expressed analysis based on the new subtypes and selected the overlapping genes for further analysis. We undertook univariate Cox analysis to analyze these genes and constructed a prognostic model by lasso regression analysis. Meanwhile, survival and gene set enrichment analyses were used to explore the prognosis and the relevant pathways, respectively. The LIRI cohort from the International Cancer Genome Consortium database was used as a reference to validate the 5mC subtypes and 5mC score.

**Results:**

Twenty-one types of 5mC regulators were employed in this study, and three 5mC-associated molecular subtypes were identified. These three subtypes presented significant differences in prognosis, immune cell infiltration, immune checkpoint inhibitors, signaling pathways, and mutational features. Compared with cluster 3, cluster 2 exhibited significantly increased expression of *PD-L1, TIM3, Galectin9, CTLA4*, and *CD80*, while *PD-L1, TIM3*, and *CD80* were higher in cluster 2 than in cluster 1. Furthermore, a 5mC-related score, composed of seven genes (*SGPP2, SALL4, B3GNT7, ROR1, MYBL2, SLC7A1*, and CAND2), was proven to be significantly associated with prognosis. The established subtypes and scores were thus successfully verified by the validated cohort.

**Conclusion:**

To the best of our knowledge, this is the first study to identify a novel molecular subtype based on 5mC regulators. The identification of the 5mC-associated subtype may help reveal the potential relation between 5mC and immunity and provide novel insights for the development of individualized therapy for HCC.

## Introduction

As one of the most common malignant tumors worldwide, hepatocellular carcinoma (HCC) has become the second leading cause of death and shows a trend of significant growth (Siegel et al., 2020). Due to the high rate of metastasis, advanced stage HCC patients usually have a poor prognosis (Ferlay et al., 2010). The occurrence and development of HCC involve interactions between genetics, epigenetics, and transcriptomic alterations (Aravalli et al., 2013). Epigenetic dysregulation promotes the initiation and progression of HCC (Chen and Wong, 2020).

As a critical type of epigenetic modification, genome methylation, including 5-methylcytosine (5mC), N6-methyladenosine (m6A), and N1-methyladenosine (m1A), has gained increasing attention recently (Roundtree et al., 2017). In mammalian cells, DNA methylation is accomplished by DNA methyltransferase enzymes, which add a methyl group at carbon-5 of the cytosine bases (Skvortsova et al., 2019), thus repressing transcription in the genome. The frequency and number of aberrant DNA methylations are thought to be closely correlated with HCC (Nishida et al., 2008). In particular, 5mC methylation has been shown to play a key role in the occurrence and development of HCC and is closely associated with clinical stages, progression, prognosis, and survival rate in HCC (Villanueva et al., 2015; Hlady et al., 2019).

Various molecular subtypes, based on different characteristics, predict the prognosis of HCC. For example, subtypes based on the immune environment have been established and verified in HCC (Li et al., 2019). In another study, an immune-based molecular classification (*PD-L1*, *IDO1*, *CTLA4*, and *CD8A*) was developed for predicting the prognosis of HCC (Xu et al., 2020). However, these molecular subtypes were far from able to meet clinical demands and more characteristic classifications need to be identified. As previously mentioned, DNA methylation, especially 5mC methylation, has a strong predictive effect on HCC. A recent study suggested that 5mC and 5hmC are dynamic markers correlated with tumor immune escape and T cell exhaustion (Xiao et al., 2020), however, the relevant mechanism remains to be explored and elucidated.

In this study, we identified novel molecular subtypes based on the gene expression of 5mC regulators. The three distinct subtypes showed remarkable differences in characteristics, including survival, gene mutations, signaling pathways, and immune response. We found that the subtypes with active 5mC methylation modification and worse prognosis may be related to the higher sensitivity of immunotherapy. In addition, based on this clustering method, we established the relevant score and verified the reliability of the subtypes. We believe that the novel molecular subtypes not only aim to elucidate the underlying association, linking 5mC methylation and immunotherapy in HCC but they can also promote the development of individualized clinical treatments.

## Materials and Methods

### Data Collection

Gene mutation and expression data, and clinical messages were retrieved from the International Cancer Genome Consortium (ICGC) database^1^ and The Cancer Genome Atlas (TCGA) database.^2^ Two independent cohorts (TCGA-LIHC, LIRI-JP) were included in our research; the TCGA-LIHC cohort was used as the training dataset and the LIRI-JP cohort was used as the validated dataset. Moreover, 21 5mC regulators were retrieved from a previous study (Chen et al., 2020), including three writers (*DNMT1*, *DNMT3A*, *DNMT3B*), 15 readers (*MBD1*, *MBD2*, *MBD3*, *MBD4*, *MECP2*, *NEIL1*, *NTHL1*, *SMUG1*, *TDG*, *UHRF1*, *UHRF2*, *UNG*, *ZBTB33*, *ZBTB38*, *ZBTB4*), and three erasers (*TET1*, *TET2*, *TET3*).

### Landscape of Genetic Variation and Unsupervised Clustering for 5mC Regulators

First, we performed a series of analyses to study the landscape of genetic variations in 5mC regulators in HCC. A summary of somatic mutations and copy number variations (CNVs) from the 21 regulators was generated using cBioPortal.^3^ The somatic mutation analysis was performed by “maftools” package and the CNV was presented by “RCircos” package. Correlation analysis among the 21 regulators was performed using the “corrplot” package.

We also explored the differences in the expression of the 21 regulators between tumor and normal groups using the Wilcoxon rank sum test. Based on the “ConsensusClusterPlus” package under the R studio software, unsupervised clustering analysis was applied to confirm distinct 5mC methylation modification patterns based on the expression of the 21 5mC regulators and classify patients for further analysis. The number of clusters and their stability was determined by a consensus clustering algorithm (Wilkerson and Hayes, 2010). Thousand repetitions were performed to guarantee the stability of the subtypes. After identifying the clusters, we performed a series of analyses to validate the established molecular subtypes. First, a principal component analysis was applied to show the distribution of samples. Next, to investigate the time-dependent prognostic value of the subtypes, survival analysis was performed using the “survival” package.

We also explored the somatic gene mutations in the different subtypes by the “maftools” package. The tumor mutational burden (TMB) was defined as the total number of errors in somatic gene coding, base substitution, gene insertions, or deletions detected in every million bases. To calculate the TMB in each case, the total number of mutations counted was divided by the exome size (38 Mb was utilized as the exome size). Tumor mutational burden correlation analysis was performed to explore the associations between TMB and the subtypes. Moreover, to investigate the differences in the biological processes in 5mC methylation modification, gene set variation analysis (GSVA) was executed using the “GSVA” package. Gene set variation analysis is usually performed to estimate the variation in the pathway and biological process activity in samples from an expression dataset (Hänzelmann et al., 2013). The gene sets of “h.all.v7.1.symbols” were retrieved from the MSigDB database^4^ for GSVA analysis. Results with a *p*-value of less than 0.05 were considered to be statistically significant.

### Immune Cell Infiltration and Immune Checkpoint Expression

We investigated the immune cell infiltration between the different 5mC related subtypes based on the CIBERSORT algorithm. CIBERSORT is a deconvolution algorithm that evaluates the proportions of 22 tumor-infiltrating lymphocyte subsets (Newman et al., 2019). The number of permutations was set to 1000, and the samples in the cohort were eligible for further investigation if they had a *p*-value < 0.05. The relevant R code can be found from the CIBERSORT website.^5^ Furthermore, eight types of immune checkpoints (Burugu et al., 2018) (*PD-1*, *PD-L1*, *TIM3*, *Galectin9*, *CTLA4*, *CD80*, *CD27*, and *CD70*) were employed to explore the underlying association between immune response and subtypes, as their receptors and ligands were both found in the gene expression matrix.

### Construction of 5mC Related Score

To identify the key genes associated with 5mC methylation in HCC, we performed a differentially expressed analysis among subtypes by the “limma” package. Both, *p*-values < 0.05 and |log^2^FC| > 1, were considered statistically significant. A volcano plot was used to visualize the differentially expressed genes, and the Venn plot was used to visualize the intersection of results. Then, we performed univariate Cox regression analysis to further investigate the prognostic genes. The genes with a *p*-value < 0.01 in univariate analysis were eligible for further selection. The lasso regression analysis was used to construct the 5mC related score by “glmnet” and “survival “package. In this analysis, a lasso penalty was used to account for shrinkage and variable selection. The optimal value of the lambda penalty parameter was defined by performing 10 cross-validations. The calculation formula for the 5mC related score was as follows:

score=(coefficientmRNA1expressionofmRNA1)

+(coefficientmRNA2×expressionofmRNA2)+⋯

+(coefficientmRNAnexpressionmRNAn)

According to the median of the established score, cases were divided into two groups (high group or low group). We also performed survival analysis and correlation analysis of the two groups. In addition, to further investigate the significantly enriched pathways in the groups, we performed the gene set enrichment analysis (GSEA). Gene set enrichment analysis is a computational method that identifies whether a previously defined set of genes shows statistically significant differences between two biological states (Mootha et al., 2003). The items of normalized *p*-value and enrichment scores were considered when choosing the most relevant pathways. Finally, univariate and multivariate Cox regression analyses were performed to verify whether the 5mC score could be an independent prognostic marker.

### Validation of Molecular Classification and Score

To validate molecular classification and score, we employed the LIRI cohort from the ICGC database. In terms of the molecular subtypes, the consensus clustering algorithm was used for 5mC related genes expression matrix and the survival analysis was executed again. Concerning the 5mC score, the gene expressions of the final included genes were extracted and used for score construction. Survival analysis was also performed for score validation.

## Results

### Landscape of Genetic Variation of 5mC Regulators in HCC

**FIGURE 1:**
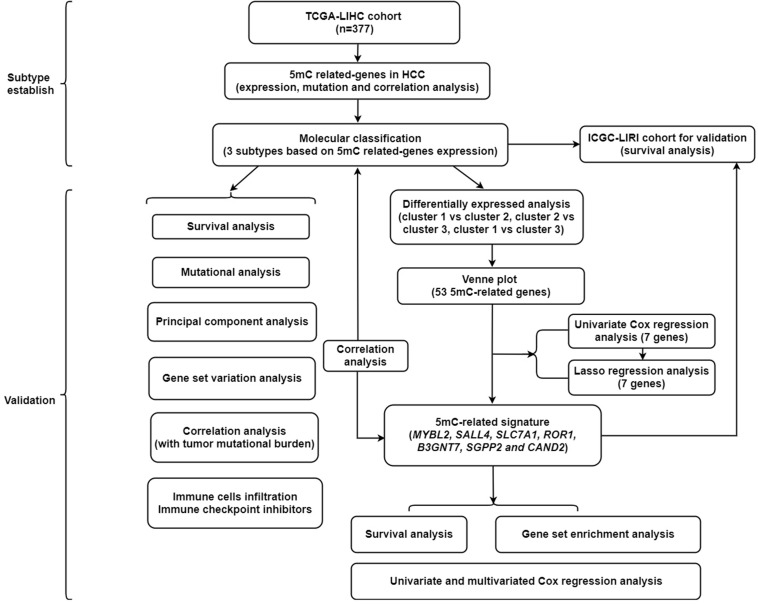
The flowchart of this study.

**FIGURE 2:**
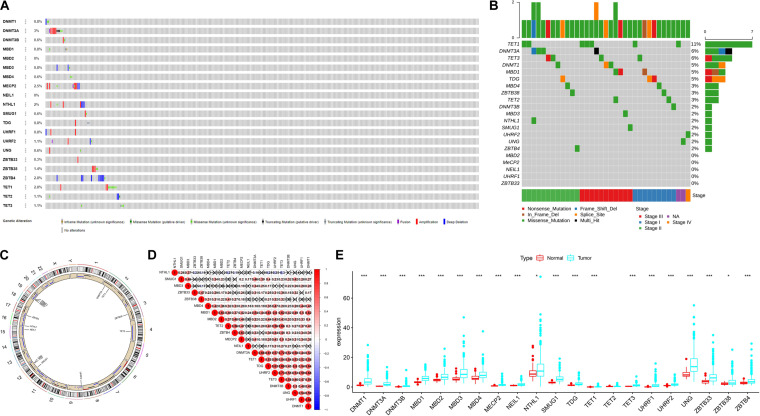
The landscape of genetic variation of 5mC regulators in HCC Panel. **(A)** Data on the somatic mutation and copy number variations of 21 5mC regulators. **(B)** Waterfall plot of somatic mutation. **(C)** Location of CNV alteration of 5mC regulators on chromosomes. **(D)** Correlation between 21 5mC regulators in HCC. **(E)** The results of differentially expressed analysis from 5mC regulators. In panel **(E)**, *p < 0.05, **p < 0.01, ***p < 0.001.

A summary of this study is presented in the form of a flowchart in Figure 1. The clinical details of the patients included in our study are summarized in Table 1. Moreover, Figure 2A provides a summary of the incidence of somatic mutations and CNV of 21 5mC regulators in HCC. In terms of the somatic mutations, among 364 cases, 36 had mutations in the 5mC regulators with a frequency of 9.89%. The results of somatic mutation (Figure 2B) showed that *TET1* presents the highest mutational frequency followed by *DNMT3A*, while the other five regulators *(MBD2*, *MECP2*, *NEIL1*, *UHRF1*, and *ZBTB33*) did not show any mutations in HCC cases. Furthermore, the location of CNV alteration in the 5mC regulators on chromosomes is shown in Figure 2C.

**TABLE 1:** Baseline patient characteristics in two cohorts.

Clinical characteristics		Number	Percent
**TCGA-LIHC (*n* = 377)**			
Survival status	Survival	249	66
	Death	128	34
Age (1 patient missing)	≤65 years	235	62.5
	>65 years	141	37.5
Gender	Female	122	68
	Male	255	32
Stage (24 patients missing)	I	175	50
	II	87	24.6
	III	86	24.4
	IV	5	1
Grade (5 patients missing)	G1	55	14
	G2	180	48
	G3	124	33
	G4	13	5
T classification (3 patients missing)	T1	185	49
	T2	95	26
	T3	81	22
	T4	13	3
**ICGC-LIRI (*n* = 260)**			
Survival status	Survival	214	82.4
	Death	46	17.6
Age	≤65 years	98	37.7
	>65 years	162	62.3
Stage	I	40	15.4
	II	117	45
	III	80	30.8
	IV	23	8.8

The correlation among 21 5mC regulators in HCC is presented in Figure 2D. It was found that the 5mC regulators not only showed a significant correlation in the same functional category but also presented a remarkable interaction among different functional categories. We also found that most of the negative correlations existed between the reader gene (*NTHL1*) and other genes (*ZBTB33*, *ZBTB38*, *TET2*, *ZBTB4*, *DNMT3A*, *TDG*, *UHRF2*, *TET3*). Based on the expression of these 5mC regulators, we investigated the mRNA expression levels of regulators between the normal and tumor groups and found that most of the regulators (except *TET2*) were significantly expressed in the tumor group (Figure 2E). The above-mentioned results demonstrated that the imbalance in the expression of 5mC regulators and cross-talk among readers, writers, and erasers may play a crucial role in HCC.

### Identification of 5mC-Associated Molecular Subtypes

**FIGURE 3:**
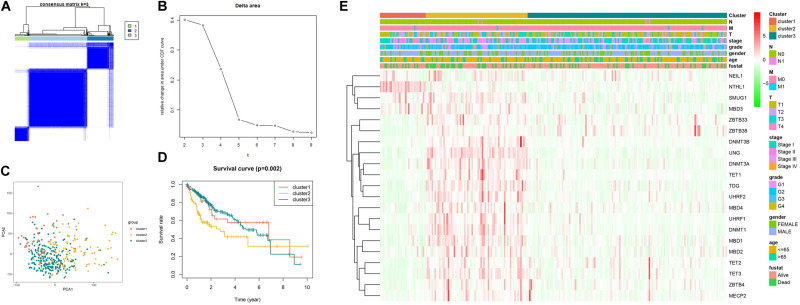
Establishment, verification, and heatmap of 5mC related molecular subtypes. Panels **(A,B)** show the most appropriate value for cluster. **(C)** Results of PCA. **(D)** Survival analysis. **(E)** Heatmap of subtypes.

As illustrated in Figure 3A, when k is equal to 3, a remarkable difference is observed between the three clusters. Figure 3B shows that the relative change was remarkable between 3 and 4. Based on the expression of the 21 5mC regulators and unsupervised clustering algorithm, the cases were divided into three clusters, including 55 cases in cluster 1, 99 in cluster 2, and 220 in cluster 3. The results of PCA, shown in Figure 3C, demonstrated that the cases from each cluster could be distinguished visually. Survival analysis for the three main 5mC methylation subtypes indicated that cluster 2 presented a survival disadvantage within five years (Figure 3D). Furthermore, according to the heatmap in Figure 3E, cluster 1 exhibited high expression of *NEIL1*, *NTHL1*, *SMUG1*, and *MBD3*, while cluster 2 was characterized by the high expression of most 5mC regulators (except for *NTHL1*), and cluster 3 showed high expression of *ZBTB33* and *ZBTB38*. We also observed that patients in cluster 2 presented a higher stage, potentially correlated to the rapid progression of HCC.

### Different Somatic Mutations in Subtypes

**FIGURE 4:**
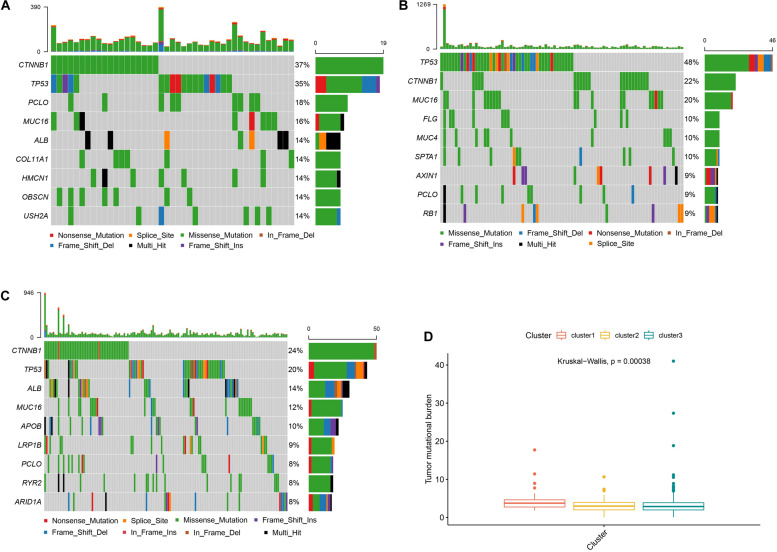
Somatic mutations and TMB in different subtypes Panels **(A–C)** show the waterfall plots of clusters 1, 2, and 3, respectively. Panel **(D)** shows the TMB correlation analysis.

As shown in Figure 4, cluster 2 presented the highest mutational rate (84/99, 84.8%) compared to cluster 1 (43/55, 78.1%) or cluster 3 (144/220, 65.4%). Meanwhile, *CTNNB1* was the most frequently mutated gene in cluster 1 or cluster 3, while *TP53* in cluster 2. We also observed significant differences between the three clusters, with cluster 1 presenting the highest TMB score.

### Relevant Pathways and Immune Checkpoints in the Different Subtypes

**FIGURE 5:**
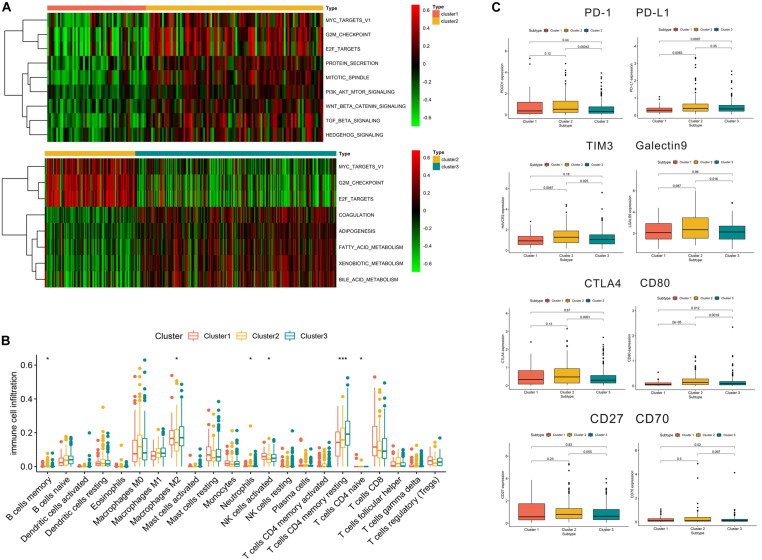
Relevant signaling pathways, immune cell infiltration, and immune checkpoints in subtypes. Panel **(A)** shows the results of gene set variation analysis. **(B)** Immune cell infiltration in the three subtypes. **(C)** Correlation between immune checkpoints and subtypes. In panel **(B)**, *p < 0.05, **p < 0.01, ***p < 0.001.

As shown in Figure 5A, we found that compared with cluster 1, cluster 2 was markedly enriched in carcinogenic activation pathways such as the TGF beta and hedgehog signaling pathways. Cluster 1 and cluster 3 presented low scores in the cell cycle pathways, such as the E2F and G2/M checkpoint pathways. Meanwhile, compared with cluster 2, cluster 3 was prominently associated with the biological process of metabolism. The subsequent analysis of immune cell infiltration demonstrated that cluster 1 significantly infiltrated the NK cells and cluster 3 significantly infiltrated the T cell CD4 memory resting (Figure 5B). Most of the significant results showed relatively lower immune cell infiltration in cluster 2. In terms of immune checkpoint inhibitors, we found that compared with cluster 3, cluster 2 exhibited significantly increased expression of *PD-L1*, *TIM3*, *Galectin9*, *CTLA4*, and *CD80*, while *PD-L1*, *TIM3*, and *CD80* had a higher expression in cluster 2 than in cluster 1 (Figure 5C).

### Construction of 5mC Related Score

**FIGURE 6:**
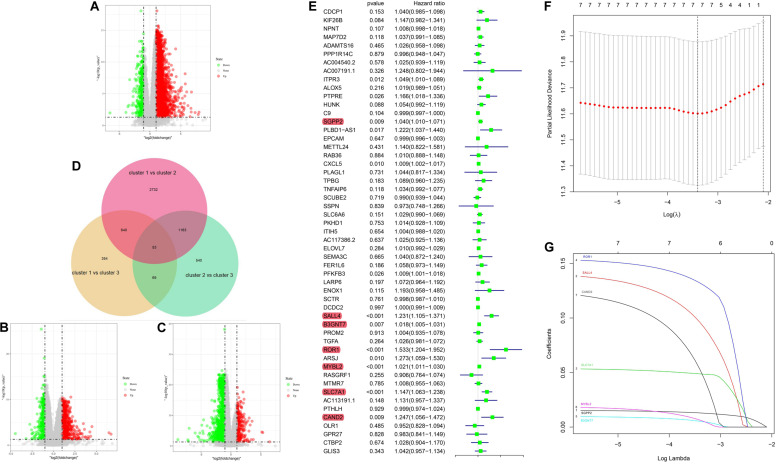
5mC score construction. Panels **(A–C)** show the volcano plots of the differentially expressed analysis. Panel **(D)** shows the Venn plot of three volcano plots. **(E)** Results of univariate Cox regression analysis. **(F,G)** Results of lasso regression analysis.

**FIGURE 7:**
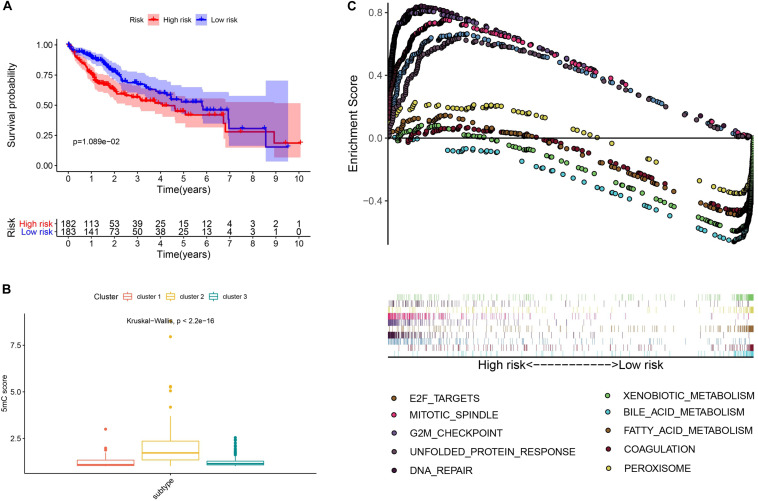
Exploration and validation of the 5mC score. Panel **(A)** shows the results of survival analysis, **(B)** shows the correlation analysis between 5mC score and subtypes, and **(C)** shows the result of gene set enrichment analysis.

**FIGURE 8:**
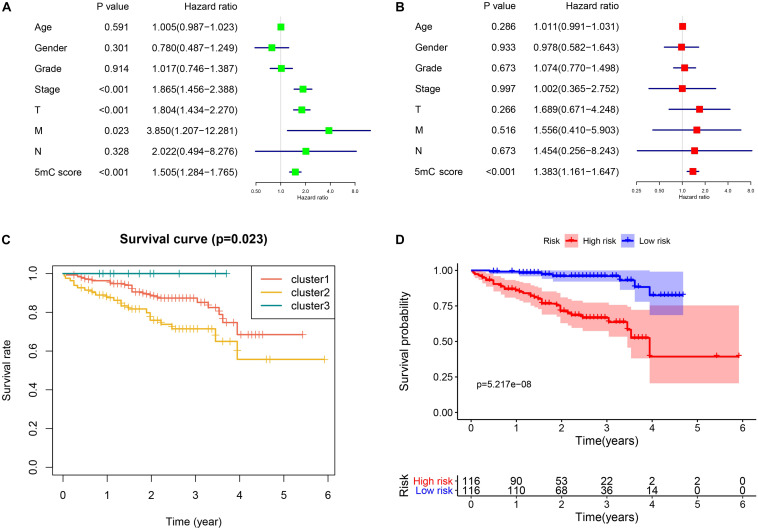
Independent biomarker analysis and validation from the ICGC cohort. Panels **(A,B)** show the results of univariate and multivariate Cox regression analysis, respectively. **(C)** Survival analysis of the subtypes. **(D)** Survival analysis of 5mC score.

To further investigate the prognostic value of 5mC regulators in HCC, we first performed a differential expression analysis of the three clusters. The volcano plots in Figures 6A–C showed significantly expressed genes. Meanwhile, the intersection of results (Figure 6D) showed that 53 genes were included for further selection. Further, the results of univariate Cox analysis (Figure 6E) and lasso regression analysis (Figure 6F,G) confirmed the score composed of seven genes, namely, *SGPP2*, *SALL4*, *B3GNT7*, *ROR1*, *MYBL2*, *SLC7A1*, and *CAND2*. The higher score presented a worse performance in overall survival analysis (Figure 7A), and cluster 2 showed a significantly higher score compared to the other two clusters (Figure 7B), which was consistent with the previous results (Figure 3D). We also observed that patients with higher scores were significantly enriched in cell cycle signaling pathways (Figure 7C), which was in accordance with cluster 2. Furthermore, the results of univariate and multivariate Cox regression analysis (Figures 8A,B) indicated that the 5mC score may serve as an independent prognostic marker in HCC.

### Verification From ICGC-LIRI Cohort

To validate the established subtype and score, we used the independent cohort (ICGC-LIRI) to perform the survival analysis. The results of survival analysis from Figure 8 showed that significant differences were found not only among the three clusters (Figure 8C) but also between the high and low score groups (Figure 8D).

## Discussion

The tumor-node-metastasis (TNM) stage system of HCC is the major current clinical standard (Liu et al., 2016). Despite its prevalence, this classification presents a low prognostic power in advanced stages (Chun et al., 2011). Several other histological subtypes, such as tumor grade, also show unsatisfactory prognostic value in HCC (Agopian et al., 2015). Consequently, there is an urgent need to establish alternative grading systems or molecular classifications for HCC. In this study, we first identified novel molecular subtypes based on 5mC regulators. The three subtypes exhibited distinct molecular characterization, and cluster 2 was of interest. While on the one hand, cluster 2 exhibited active 5mC methylation, on the other, it was mainly enriched in cell cycle-related pathways consistent with poor prognosis. Interestingly, we found that the advanced stage and poor prognosis of cluster 2 may present a high sensitivity of immunotherapy.

As eraser regulators in 5mC methylation, ten-eleven translocation enzymes (*TET1*, *TET2*, and *TET3*) are often downregulated in cancer-inducing DNA demethylation by converting 5mC to 5-hydroxymethylcytosine (5hmC). It has been verified that high *TET1* expression is associated with low levels of immune and defense response markers indicating that the prognostic value of *TET1* expression in cancer might be related to the immune status of tumors (Collignon et al., 2018). In terms of the family of DNMTs, as the writer regulators in 5mC methylation, both *DNMT1* and *DNMT3A* have been shown to regulate T cell differentiation and function (Ladle et al., 2016; Youngblood et al., 2017). Meanwhile, 5mC and 5hmC are considered to be dynamic markers correlated with tumor immune evasion and potential biomarkers of response to cancer immunotherapy (Xiao et al., 2020).

TMB is considered to be a biomarker that contributes to the expression of neoantigens and is positively correlated with the response of immune checkpoint inhibitors (Chan et al., 2019). In clusters 2 and 3, the patients presented with relatively low TMB, however, they carried several different genetic mutations. A previous study demonstrated that CTNNB1 mutation induced the upregulation of β-catenin expression and increased the activity of the Wnt pathway (Javanmard et al., 2020). The upregulation of c-Myc in the Wnt pathway leads to proto-oncogenic effects in liver malignancies (Kim et al., 2008). The TP53 mutations in HCC patients in western countries were correlated with worse prognosis (Takai et al., 2014). At present, a number of studies have found a robust correlation between genetic mutation and poor prognosis of HCC. However, whether genetic mutations correlate with immunologic protein expression remains to be further investigated. In this study, we speculated that different genetic mutations that manifest as differences in TMB may correlate with immune modulation. Although cluster 2 presented a relatively low level of immune cell infiltration, we considered the binding of immune checkpoints and its receptors to be of interest. Due to the higher expression of immune checkpoints in cluster 2, we speculated that patients in cluster 2 may be more sensitive to immunotherapy than the other two clusters.

In particular, we observed that the *TIM3* (*HAVCR2*)-*Galectin9* (*LGALS9*) and the *CTLA4-CD80* interactions were highly expressed and may be activated in cluster 2. *LGALS9* is the ligand that interacts with *HAVCR2* (Zhu et al., 2005). *HAVCR2* is an inducible NK cell receptor that enhances interferon gamma production in response to *LGALS9* (Gleason et al., 2012). *CTLA4* is one of the first inhibitory receptors that plays a role in the suppression of T-cell responses (Linsley et al., 1991). *CTLA4* interacts with *CD80*/*CD86*, thereby limiting T-cell activation and leading to anergy (Dyck and Mills, 2017). Therefore, the molecular and immune characteristics in cluster 2 elucidated the intrinsic association between 5mC methylation modification and immunotherapy in HCC. Although immune checkpoint inhibitors have been shown to affect the treatment of certain cancers, they are related to significant and frequent adverse events, such as colitis, skin disorders, and hepatitis (Callahan et al., 2016). As a result, it is necessary to investigate biomarkers to identify whether patients might be sensitive to immune checkpoint blockade treatment. Based on the molecular features of cluster 2, HCC patients with active 5mC methylation may benefit from immune checkpoint blockade treatment. In other words, 5mC methylation modification could further limit the application of immunotherapy and promote individualized treatment in HCC.

Considering the individual heterogeneity of 5mC methylation modification, it was necessary to quantify the 5mC methylation of different tumors. We established a novel scoring system to evaluate 5mC methylation modification in patients with HCC. The 5mC methylation modification characterized by cluster 2 presented a high 5mC score. Higher 5mC scores lead to activation of tumor progression pathways and worse prognosis consistent with the molecular features of cluster 2. The molecular subtype and relevant scores were validated in another independent cohort, suggesting that 5mC methylation modification is a reliable and robust tool for the comprehensive assessment of HCC.

A previous study (Cancer Genome Atlas Research Network, 2017) identified three subtypes based on DNA copy number, DNA methylation, RNA, miRNA, and proteomic expression in HCC cases. Our research confirmed three subtypes based on the genetic expression of 5mC regulators and emphasized the specific biological function (5mC methylation) in HCC. Compared with the above-mentioned study, we exhibited 5mC methylation characterization using the genetic expression, presenting a comprehensive genomic characterization that has not been reported previously. In another study by Zhang et al. (2019), the novel immunophenotypic classification of HCC was identified by the specific marker gene expression levels, which aimed to investigate the immunologic characterization of HCC cases. However, they did not explore the correlation between immunity and methylation. Our study found different immunologic landscapes between different 5mC methylation activations. Thus, the findings in our study link 5mC methylation and immunity in HCC, which may further help in developing novel clinical immunologic treatments.

To the best of our knowledge, this is the first study to identify 5mC-associated molecular subtypes in HCC. In contrast to a previously published study (He et al., 2020), our research revealed the underlying association between 5mC methylation and immunotherapy in HCC. Moreover, a greater number of 5mC regulators included and the comprehensive methodology used in our research enabled the identification of a reliable score. Furthermore, we proposed the potential mechanism of immune modulation in patients with active 5mC methylation, which may contribute to further functional experiments and clinical trials.

There are some limitations to our study. First, no specific molecular features were observed in cluster 1 or cluster 3, which may be attributed to the small sample size. Further investigation and definition of other clusters may aid a deeper understanding of the effects of 5mC methylation in HCC. Second, we cannot further explore the immunotherapeutic response between 5mC associated molecular subtypes because no related cohort was performed in HCC. Third, the intrinsic weakness of genetic heterogeneity may affect our results and influence further clinical applications. Finally, using a bioinformatics approach, our research only focused on the genetic expression of the presented genes, while the protein expression remains to be validated.

## Conclusion

To the best of our knowledge, this is the first study to identify novel molecular subtypes based on 5mC regulators that may help reveal the potential relationship between 5mC methylation and immunity and provide novel insights for developing individualized therapy for HCC.

## Data Availability Statement

Publicly available datasets were analyzed in this study. This data can be found here: TCGA: LIHC (https://portal.gdc.cancer.gov/); ICGC: LIRI (https://dcc.icgc.org/).

## Author Contributions

ZM and SZ designed the manuscript. ZM, ZC, and SL wrote and completed the manuscript. ZM, SL, and YC completed the data download and analysis. All the authors approved the final manuscript.

## Conflict of Interest

The authors declare that the research was conducted in the absence of any commercial or financial relationships that could be construed as a potential conflict of interest.

## Footnotes

1 https://dcc.icgc.org/

2 https://portal.gdc.cancer.gov/

3 https://www.cbioportal.org/

4 https://www.gsea-msigdb.org/gsea/index.jsp

5 https://cibersortx.stanford.edu/
